# Supplementary figures and images for: Dysbiotic oral microbiota and infected salivary glands in Sjögren’s syndrome
Source: PLoS One. 2020 Mar 24;15(3):e0230667. doi: 10.1371/journal.pone.0230667 (PMC7092996; doi:10.1371/journal.pone.0230667)

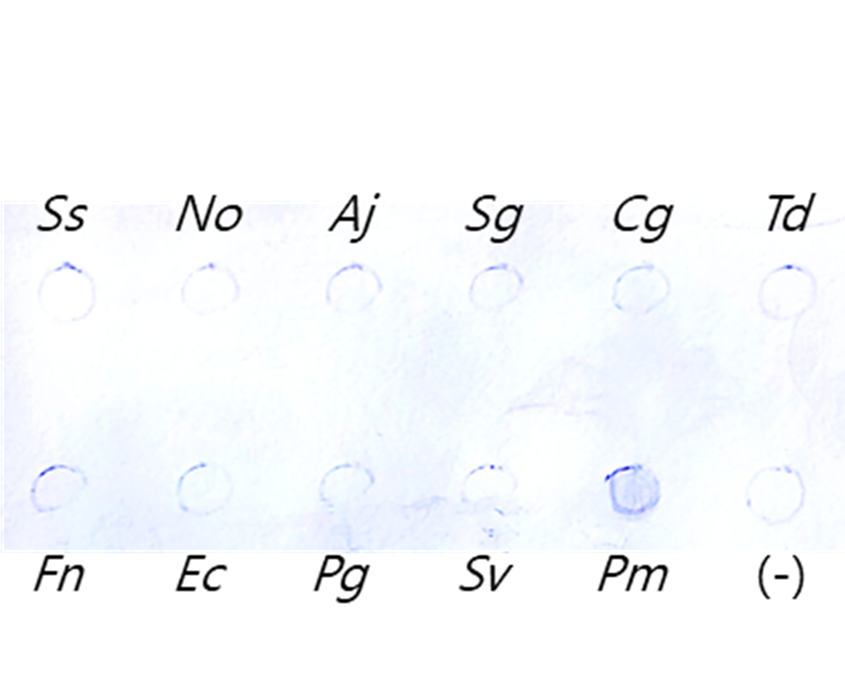

Supplement: S1 Fig — The lysates of Streptococcus salivarius (Ss), Neisseria oralis (No), Acinetobacter johnsonii (Aj), Streptococcus gordonii (Sg), Capnocytophaga gingivalis (Cg), Treponema denticola (Td), Fusobacterium nucleatum (Fn), Porphyromonas gingivalis (Pg), Saccharomonospora viridis (Sv), and Prevotella melaninogenica (Pm) were prepared. After measuring the DNA concentration, each bacterial lysate that contains 100 ng DNA was blotted onto a nylon membrane. The membrane was then hybridized with the P. melaninogenica-specific probe. (TIF) [file pone.0230667.s001.TIF]

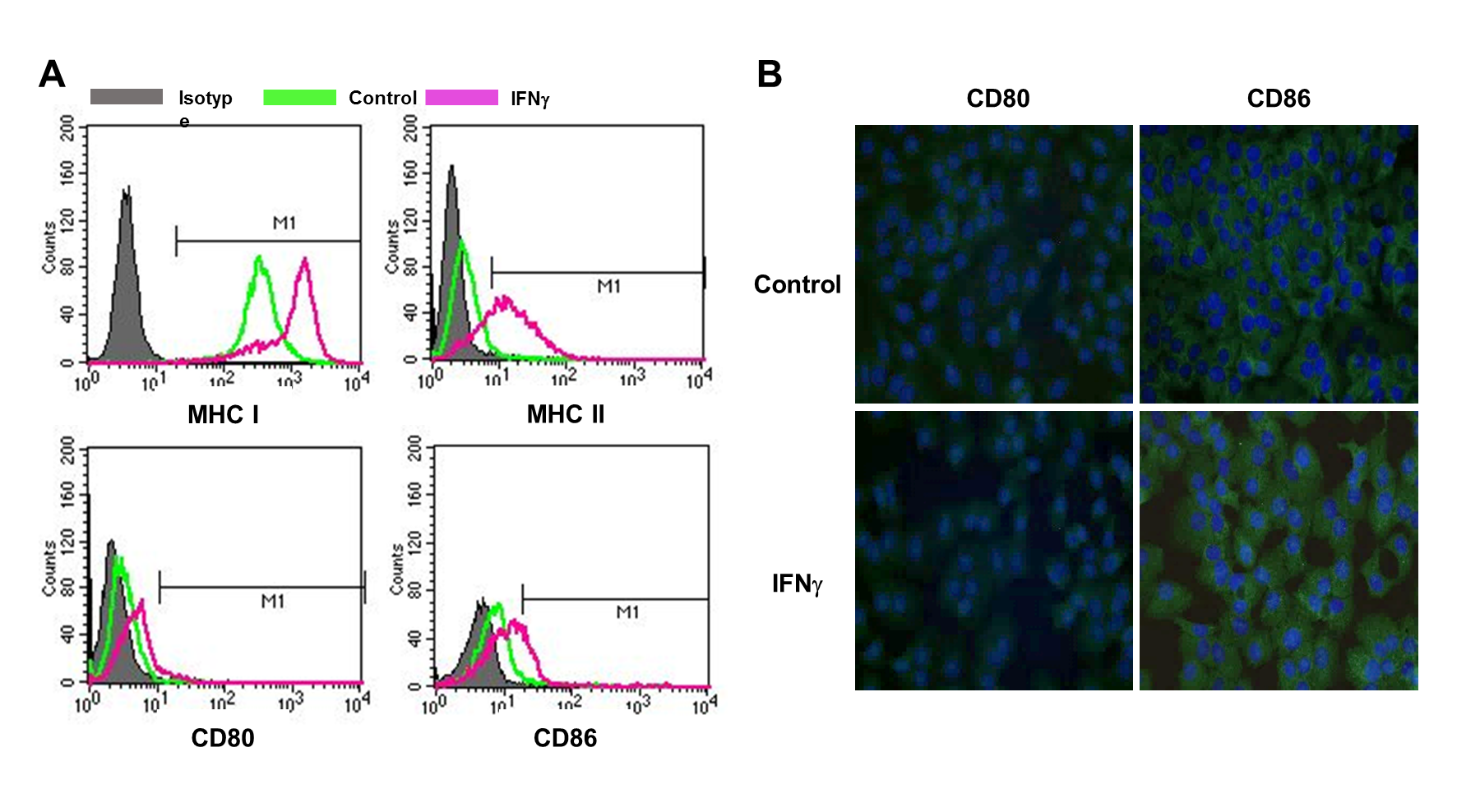

Supplement: S2 Fig — HSG cells (4 × 10^4^ cells/well) were plated into 24-well plates and treated with IFNγ for 72 hours. The expression of APC-related surface molecules on HSG cells was analyzed by flow cytometry and fluorescence microscopy. (A) Upregulation of APC markers such as MHC I, MHC II, and costimulatory molecules on HSG cells was analyzed by flow cytometry. (B) The expression of costimulatory molecules (green) on HSG cells in the absence or presence of IFNγ was confirmed by fluorescence microscopy. (TIF) [file pone.0230667.s002.TIF]

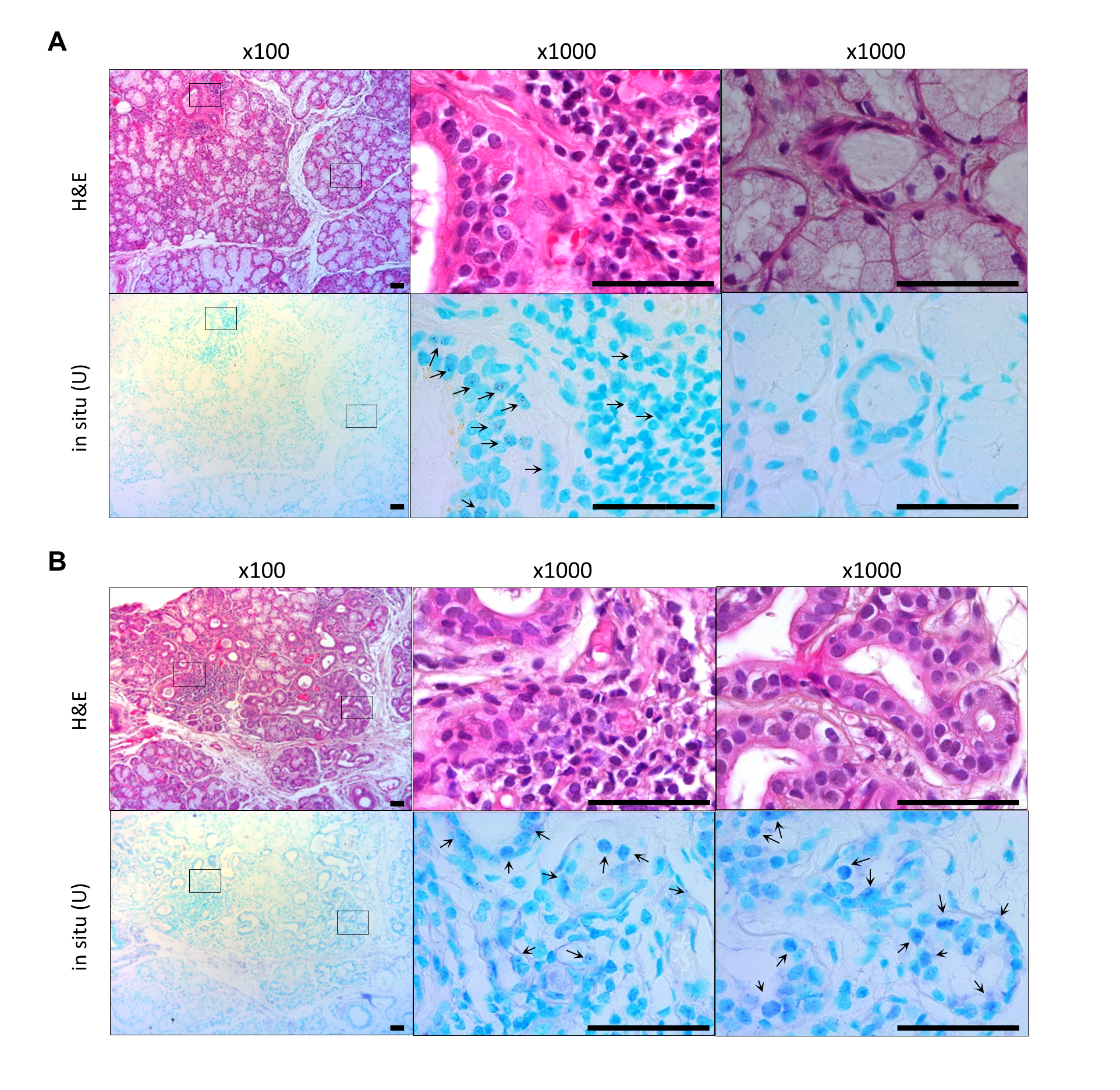

Supplement: S3 Fig — The sections of paraffin-embedded LSG tissues obtained from Control subjects or SS patients were subjected to H&E stain and in situ hybridization (ISH) using a universal (U) probe. (A) LSG with non-specific chronic inflammation from Control subjects. (B) LSG with FLS > 1 from SS patients. The areas marked with a square were examined with higher magnification. Arrows indicated infected ductal cells. Scale bars indicate 50 μm. (TIF) [file pone.0230667.s003.TIF]
