# Supplementary material for: Dysbiotic oral microbiota and infected salivary glands in Sjögren’s syndrome
Source: PLoS One. 2020 Mar 24;15(3):e0230667. doi: 10.1371/journal.pone.0230667 (PMC7092996; doi:10.1371/journal.pone.0230667)
Supplement: S1 Table — (DOCX) [file pone.0230667.s004.docx]

**S1 Table. The list of medications**

|  | UWSFR (ml/min) | Medication |
| --- | --- | --- |
| SC1 | 0.03 | Trazodone, Bisphosphonate^†^ |
| SC2^*^ | 0.1 | None |
| SC3 | 0.05 | Ticagrelor^†^, Acetylsalicylic acid, Simvastatin^†^ |
| SC4 | 0.06 | Amlodipine^†^, Metformin^†^, Simvastatin^†^, Zolpidem, Chlorthalidone |
| SC5 | 0.05 | Alprazolam, Zolpidem, Naproxen^†^ |
| SC6 | 0.02 | Metformin^†^, Simvastatin^†^, Pantoprazole^†^, Zolpidem |
| SC7 | 0.07 | Oxcarbazepine, Acetylsalicylic acid^†^, Acetyl-L Carnitine^†^, Duloxetine^†^, Escitalopram, Alprazolam, Risperidone, Cimetidine, Tianeptine |
| SC8 | 0.06 | Propranolol^†^, Benzodiazepines^†^, Bupropion, Itopride, Cyanocobalamin/Cyproheptadine/DL carnitine^†^ complex |
| SC9 | 0.05 | Ranitidine^†^, Clotiazepam, Mosapride citrate |
| SC10 | 0.02 | Amlodipine^†^/Losartan complex, Acetaminophen^†^, Duloxetine^†^, Alprazolam |

UWSFR: unstimulated whole salivary flow rate

^*^Recently received radiation therapy and chemotherapy for cervical cancer

^†^Medications with known antimicrobial activity to any bacterial species are underlined.
