# Supplementary material for: Dysbiotic oral microbiota and infected salivary glands in Sjögren’s syndrome
Source: PLoS One. 2020 Mar 24;15(3):e0230667. doi: 10.1371/journal.pone.0230667 (PMC7092996; doi:10.1371/journal.pone.0230667)
Supplement: S2 Table — (DOCX) [file pone.0230667.s005.docx]

**S2 Table. Genera^a^ associated with Control vs. SS**

| Name | Median (Range) | | *P*^b^ | Median (Range) | | *P*^b^ |
| --- | --- | --- | --- | --- | --- | --- |
|  | Control w/o dryness | SS w/o dryness |  | Control dryness | SS dryness |  |
| *Streptococcus* | 42.16 (82.50) | 59.91 (34.63) | 0.096 | 65.79 (55.92) | 71.96 (46.35) | 0.460 |
| *Neisseria* | 17.47 (45.96) | 11.45 (35.22) | 0.826 | 8.51 (51.09) | 0.06 (4.42) | **0.002** |
| *Haemophilus* | 8.15 (33.48) | 4.35 (10.12) | 0.232 | 1.21 (13.35) | 0.60 (13.81) | 0.670 |
| *Prevotella* | 2.35 (13.48) | 4.94 (17.68) | 0.082 | 0.94 (16.06) | 3.52 (19.76) | 0.360 |
| *Fusobacterium* | 1.81 (22.80) | 0.77 (2.54) | 0.082 | 0.91 (1.67) | 0.50 (4.21) | 0.624 |
| *Porphyromonas* | 0.33 (4.09) | 0.46 (2.31) | 1.000 | 0.23 (1.63) | 0.05 (1.81) | 0.072 |
| *Leptotrichia* | 0.50 (3.62) | 0.10 (0.68) | 0.034 | 0.20 (2.88) | 0.07 (1.28) | 0.190 |
| *Lautropia* | 0.18 (6.46) | 0.03 (0.21) | 0.040 | 0.01 (2.31) | 0.00 (0.24) | **0.004** |
| *Lactobacillus* | 0.00 (0.35) | 0.00 (0.02) | 1.000 | 0.00 (0.02) | 0.04 (3.60) | 0.024 |
| *Campylobacter* | 0.28 (2.76) | 0.03 (0.13) | 0.002 | 0.05 (0.54) | 0.02 (0.25) | 0.196 |
| *Capnocytophaga* | 0.17 (0.83) | 0.08 (0.25) | 0.304 | 0.08 (2.12) | 0.01 (0.06) | **0.004** |
| *Corynebacterium* | 0.13 (1.75) | 0.11 (0.56) | 1.000 | 0.04 (0.61) | 0.02 (0.66) | 0.198 |
| *Atopobium* | 0.07 (0.90) | 0.13 (0.87) | 0.988 | 0.07 (0.56) | 0.21 (1.22) | 0.120 |
| *Lachnoanaerobaculum* | 0.15 (0.35) | 0.12 (0.23) | 0.990 | 0.12 (0.60) | 0.04 (0.29) | 0.064 |
| *Saccharimonas* | 0.11 (1.32) | 0.01 (0.08) | 0.022 | 0.02 (0.44) | 0.00 (0.39) | 0.884 |
| *Halomonas* | 0.00 (2.12) | 0.00 (0.00) | 0.126 | 0.00 (0.17) | 0.00 (0.00) | 0.428 |
| *Kingella* | 0.10 (0.79) | 0.07 (0.19) | 0.904 | 0.03 (0.18) | 0.00 (0.23) | 0.304 |
| *Moryella* | 0.07 (0.35) | 0.02 (0.16) | 0.096 | 0.03 (0.22) | 0.00 (0.27) | 0.238 |
| *AF432141_g* | 0.00 (1.32) | 0.00 (0.00) | 0.074 | 0.00 (0.04) | 0.00 (0.00) | 0.146 |
| *Treponema* | 0.00 (0.76) | 0.00 (0.00) | 0.042 | 0.01 (0.13) | 0.00 (0.11) | 0.102 |
| *AM420062_g* | 0.01 (0.45) | 0.00 (0.13) | 0.386 | 0.00 (0.14) | 0.00 (0.20) | 0.426 |
| *Tannerella* | 0.01 (0.76) | 0.00 (0.05) | 0.150 | 0.01 (0.03) | 0.00 (0.04) | 0.248 |
| *Treponema_g1* | 0.02 (0.71) | 0.00 (0.03) | 0.046 | 0.00 (0.17) | 0.00 (0.00) | 0.018 |
| *Eikenella* | 0.03 (0.19) | 0.02 (0.09) | 1.000 | 0.01 (0.07) | 0.00 (0.05) | **0.004** |
| *Staphylococcus* | 0.00 (0.11) | 0.00 (0.00) | 0.074 | 0.01 (0.05) | 0.00 (0.32) | 0.188 |
| *Neisseriaceae_uc* | 0.01 (0.09) | 0.02 (0.08) | 1.000 | 0.02 (0.04) | 0.00 (0.00) | **0.000** |
| *Pasteurellaceae_uc* | 0.01 (0.17) | 0.00 (0.06) | 0.232 | 0.00 (0.03) | 0.00 (0.04) | 0.808 |
| *Cardiobacterium* | 0.00 (0.27) | 0.00 (0.01) | 0.606 | 0.00 (0.05) | 0.00 (0.00) | 0.146 |
| *Johnsonella* | 0.00 (0.34) | 0.00 (0.01) | 1.000 | 0.00 (0.09) | 0.00 (0.00) | 0.052 |
| *Propionibacterium* | 0.00 (0.06) | 0.00 (0.03) | 0.716 | 0.01 (0.05) | 0.00 (0.07) | 0.044 |
| *Simonsiella* | 0.00 (0.08) | 0.00 (0.00) | 0.072 | 0.00 (0.13) | 0.00 (0.00) | 0.428 |
| *AM419986_g* | 0.00 (0.05) | 0.00 (0.02) | 0.234 | 0.00 (0.08) | 0.00 (0.00) | 0.052 |
| *Eubacterium_g14* | 0.00 (0.12) | 0.00 (0.00) | 0.340 | 0.00 (0.06) | 0.00 (0.04) | 0.106 |
| *Veillonellaceae_uc* | 0.01 (0.06) | 0.00 (0.01) | 0.092 | 0.00 (0.02) | 0.00 (0.02) | 1.000 |
| *Eubacterium_g15* | 0.00 (0.05) | 0.00 (0.000 | 0.074 | 0.00 (0.01) | 0.00 (0.05) | 0.716 |
| *AM420048_g* | 0.00 (0.09) | 0.00 (0.000 | 0.126 | 0.00 (0.00) | 0.00 (0.00) | 1.000 |
| *Phreatobacter* | 0.00 (0.07) | 0.00 (0.00) | 0.210 | 0.00 (0.02) | 0.00 (0.00) | 0.146 |
| *AM420052_g* | 0.00 (0.03) | 0.00 (0.00) | 0.126 | 0.00 (0.02) | 0.00 (0.01) | 0.064 |
| *AM420198_g* | 0.00 (0.05) | 0.00 (0.010 | 1.000 | 0.00 (0.06) | 0.00 (0.00) | 0.052 |

^a^Genera that are significantly associated with Control or SS by the Mann-Whitney and Benjamini–Hochberg tests are listed.

^b^By Kruskal-Wallis test with Boneferroni adjustment for post-hoc. Bolded values also passed the Benjamini–Hochberg test.
