# Supplementary material for: Dysbiotic oral microbiota and infected salivary glands in Sjögren’s syndrome
Source: PLoS One. 2020 Mar 24;15(3):e0230667. doi: 10.1371/journal.pone.0230667 (PMC7092996; doi:10.1371/journal.pone.0230667)
Supplement: Attachment — Submitted filename: Responses to Reviewer.docx [file pone.0230667.s006.docx]

We appreciate reviewers for their time and helpful comments that improved the quality of this manuscript.

Reviewer #1:
Minor comments
While the intestinal microbiota in SS patients and other disorders is an interesting avenue of research, it seems of no connection to the study describing only the oral microbiota. Especially since, in the case of diseases listed in line 66, oral microbiota is also being investigated, particularly with the connection to periodontitis.

→ As the reviewer suggested, intestinal microbiota has been removed, and the sentence has been edited with an appropriate reference as following (lines 64-66): A growing body of evidence suggests that dysbiosis of the oral microbiome is associated with the pathogenesis of several autoimmune diseases, such as systemic lupus erythematosus, Crohn's disease, and rheumatoid arthritis. [9].

It is unclear what the numbers in brackets in Tables 2-4 next to relative abundance of each bacteria species represent.

→ Table legends have been added as following: ^a^Expressed as the median (range)

Some of the medications listed in S1Table are known to have bactericidal activity (such as simvastatin).

→ Thank you for pointing out the antibacterial activity of non-antibiotic drugs. Frankly speaking, we did not know it. In Discussion section, this issue has been addressed as following (lines 371-377): It is noteworthy that antimicrobial activities have been observed by a number of non-antibiotic drugs such as the beta-adrenergic receptor antagonists, diuretic drugs, H1 antihistamines, psychotropic drugs, non-steroid anti-inflammatory drugs, and proton pump inhibitors [30,31], and some of the medications taken by the drug-related sicca patients are known to have antibacterial activity (S2 Table). Although the control subjects without or with dryness harbored comparable levels of total bacterial loads, the medications with antimicrobial activity may have affected the composition of oral microbiota.

Throughout the manuscript, the authors state that "bacterial species was increased..." or "genera was decreased". Do the authors mean the abundance of a particular species?
→ “the relative abundance of” was put before the name of taxa, or “enriched” and “underrepresented” have been used instead of “increased” and “decreased”, respectively.

The results presented in Fig. 2 were analysed with t-test. Why did the authors choose that as opposed to the one-way analysis of variance (ANOVA)?
→ Because we wanted to know if each species can induce the APC-like phenotype compared with non-infected control, not the comparison of the effects of different species.

It is unclear which studies the authors refer to when discussing "previous studies" in line 331 in the Discussion section. Salivary microbiota in SS has already been somewhat investigated and it could benefit the current manuscript to draw a more in-depth comparison with, for example, most recent reports (DOI): 10.1080/20002297. (referenced in the introduction of this manuscript)
→ Reference citation [10,11,13,14] has been added (line 337). In addition, a paragraph that compared the oral microbiota data with previous studies has been added (lines 348-356).

Please clarify or modify the following sentences.
Line 80: All subjects were female because there were only four male SS patient in our cohort.

→ All subjects were female because male SS patients are very rare in Korea. (Line 92)

Line 97: All biopsies were from Asian female obtained at the time of diagnosis.

→ We found out that participation to the SICCA study was not based on treatment or non-treatment. Therefore, “obtained at the time of diagnosis” has been removed from the sentence. (line 100)

Line 334 - 336: The salivary microbiota of individuals with dental caries is characterized with a reduced Shannon diversity, which may be attributed to decreased salivary pH. Thus, the increased Shannon diversity in the previous studies may reflect increased caries prevalence in SS. [ref?]

→ The sentence has been changed as following (line 340): Thus, the decreased Shannon diversity in the previous studies may reflect increased caries prevalence in SS.

Line 339: Interestingly, the shannon index had a weak but significant positive correlation with total bacterial load that was also significantly increased in SS.

→ Line 345: Interestingly, the Shannon index had a weak but significant positive correlation with total bacterial load that was also significantly increased in SS compared to control.

Line 382: Since bacteria were detected within ductal cells and the area of infiltration with any type of inflammation, (...).

→ Line 404-405: Since bacteria were detected within ductal cells and the area of infiltration with not only FLS but also nonspecific/sclerosing chronic inflammation, bacterial infection might be the result of barrier disruption caused by inflammation and/or fibrosis.

Please check abbreviations.
→ Editorial errors regarding the use of abbreviations have been corrected.

Reviewer #2 Comments:

1.In baseline characteristics (Table 1) , please check if age of controls and ss without dryness and corresponding p value is correct.
→ Yes, it’s correct by ANOVA with Bonferroni post hoc test.

2. Could the fold changes of microbiome between SS and controls also be provided? This would allow for comparison with previous studies
→ Statistically differently distributed taxa between SS (n=25) and controls (n=25) are already presented at the phylum (Fig. 1E), genera (S2 Table), and species levels (Table 2). Because the relative abundance of SS (n=25) and controls (n=25) groups for each genera are not shown in the S2 Table, the names of 5 genera enriched in SS have been listed in the result section line 195 as following (line 195): At the genus level, 5 genera, including *Streptococcus*, *Prevotella*, *Lactobacillus*, *Atopobium*, and *Staphylococcus*, were enriched but 34 genera were underrepresented in SS compared to control, among which 9 and 8 genera maintained significant differences in non-dry and dry conditions, respectively (S2 Table).

3.In contrast to at least 9 previous studies on oral microbiome in pSS, they have reported increase in diversity. In discussion, the authors state that this could be attributed to decrease in salivary gland defensins reported by Kaneda et al. However, proteomic studies have reported increase in antimicrobial peptides such as lactoferrin, lysozyme, defensin (Ryu et al Rheumatology 2016, Peluso et al Arthritis Rheum 2007)
→ Proteomics studies used the same volume of saliva for analysis. The majority of differently expressed proteins are up-regulated in SS patients (Cecchettini et al. Clinical Proteomics 2019;16:26), but this, in a way, reflects concentration of proteins due to the reduced salivary flow rate in SS patients. Furthermore, many PRPs, statherin, and Histatins that have a role in agglutination of bacteria and generation of antimicrobial peptides were decreased in the saliva of SS (Peluso et al Arthritis Rheum 2007). Therefore, we left the citation of decreased expression of human beta-defensins in the salivary gland of SS patients.

4. Also none of the previous studies have reported an increase in Prevotella. In fact two studies Siddiqui et al(Journal of microbiology 2016) and Rusthen et al (Plos one 2019) report increase Prevotella in controls. It is suggested that the authors discuss the prior microbiome studies in pSS.
→ Considering the differences in the sampling sites (whole mouthwash, saliva, tongue, and buccal mucosa), ethnicity, diet, and the sequenced V regions of 16S rRNA gene, the results of oral microbiota analysis cannot be directly compared with those of previous studies. For example, Meulen et al. [14] reported lower Streptococcus relative abundances in pSS compared with HCs, while ref 11-13 and our results showed higher oral Streptococcus relative abundance in patients with pSS compared with HCs. One previous study by Li et al [12] reported increased Prevotella in SS. The increased relative abundance of Prevotella in controls reported by Siddiqui et al(Journal of microbiology 2016) and Rusthen et al (Plos one 2019) were not significantly different from those in SS. We added a paragraph in Discussion section that addressed the changes in the bacterial composition in comparison with previous works as following: Considering the differences in the sampling sites (whole mouthwash, saliva, tongue, and buccal mucosa), ethnicity, diet, and the sequenced V regions of 16S rRNA gene, the results of oral microbiota analysis cannot be directly compared with those of previous studies. Nevertheless, there are several common findings. Increased relative abundances of phylum Firmicutes [10,13] and genera *Streptococcus* [11-13], *Lactobacillus* [10,12], *Prevotella* [12], and *Atopobium* [10] but decreased relative abundances of phyla Proteobacteria [10,12] and Spirochaetes [13] and genera *Neisseria* [10,12], *Haemophilus* [10,12], *Leptotrichia* [11,12], *Fusobacterium* [11], *Lautropia* [10], *Porphyromonas* [12,13], *Treponema* [13], and *Tannerella* [12,13] in SS compared to those in controls have been repeatedly observed, including within the current study. The reduced relative abundance of *P. gingivalis* also agrees with previous studies [11, 13] and explains the lack of increased risk of periodontitis in SS patients, despite the reduced salivary antimicrobial function [28, 29].

Minor comments

Page 19 line 335 in place of increased Shannon Index , the authors have intended to mention decreased- possibly typo

→ Thanks for pointing out this mistake. It has been corrected.
