# Supplementary material for: Dysbiotic oral microbiota and infected salivary glands in Sjögren’s syndrome
Source: PLoS One. 2020 Mar 24;15(3):e0230667. doi: 10.1371/journal.pone.0230667 (PMC7092996; doi:10.1371/journal.pone.0230667)
Supplement: Attachment — Submitted filename: Responses to Reviewer2.docx [file pone.0230667.s007.docx]

We appreciate reviewers for their time and helpful comments that improved the quality of this manuscript.

Reviewer #2: A number of recent papers on Sjogren's saliva microbiome which appeared in the recent years have not been cited.

→ Two papers published in 2019 were additionally cited in Introduction (Page 4 line 1-3) and Discussion (Page 19 line 20) sections.

16.	Sharma D, Sandhya P, Vellarikkal SK, Surin AK, Jayarajan R, Verma A, et al. Saliva microbiome in primary Sjogren's syndrome reveals distinct set of disease-associated microbes. Oral diseases. 2019. Epub 2019/09/13. doi: 10.1111/odi.13191. PubMed PMID: 31514257.

17.	Sembler-Møller ML, Belstrøm D, Locht H, Enevold C, Pedersen AML. Next-generation sequencing of whole saliva from patients with primary Sjögren’s syndrome and non-Sjögren’s sicca reveals comparable salivary microbiota. Journal of Oral Microbiology. 2019;11(1):1660566. doi: 10.1080/20002297.2019.1660566.
